# Recent Advances in Single-Cell Analysis of Atherosclerotic Plaque Biology

**DOI:** 10.31662/jmaj.2025-0397

**Published:** 2025-12-12

**Authors:** Yusuke Adachi, Alyssa Grogan, Rika Kawakami, Tatsuya Shiraki, Teruo Sekimoto, Takamasa Tanaka, Kazuhiro Fujiyoshi, Takafumi Nakayama, Tomoyo Hamana, Desiree Williams, Keisha Medina Diaz, Renu Virmani, Aloke V. Finn

**Affiliations:** 1 CVPath Institute, Inc., Gaithersburg, MD, USA

**Keywords:** atherosclerosis, atherosclerotic plaques, single-cell transcriptomics, spatial transcriptomics, macrophages, T cells

## Abstract

Atherosclerosis, the leading cause of coronary artery disease, stroke, and peripheral arterial disease, is now recognized as a lipid-driven disease complicated by an immune response that plays a major role in its pathogenesis. The response-to-injury hypothesis proposed by Ross et al. laid the foundation for understanding atherosclerosis as a chronic inflammatory process, in which endothelial injury and lipid insudation trigger immune activation, smooth muscle cell proliferation, and plaque formation. Traditional approaches, such as immunohistochemistry, flow cytometry, and bulk RNA sequencing, have identified macrophages and T cells as the key immune players in plaques. However, these methods lack the resolution to differentiate among diverse immune cell states or to detect rare but functionally significant populations. Recent advances in single-cell and spatial transcriptomic technologies have revolutionized our understanding of atherosclerotic plaques. These methods have generated detailed cellular atlases in murine models and human atherosclerotic tissues, revealing previously unrecognized immune cell subsets and novel pathogenic pathways. Single-cell analyses have identified a heterogeneous spectrum of macrophages, including resident-like, inflammatory, and TREM2^high^ foamy subsets, in addition to a CD163^+^ macrophage subset, including the hemoglobin-stimulated macrophage [M(Hb)] phenotype. In parallel, functionally diverse T-cell subsets with specialized pro- and anti-inflammatory roles have also been characterized. Spatial transcriptomics has provided further insights into the anatomical organization of these immune populations within plaques, highlighting region-specific inflammatory niches and fibrous-cap dynamics. Furthermore, single-cell T-cell receptor sequencing has identified antigen-specific T-cell expansions, supporting the hypothesis that atherosclerosis exhibits autoimmune-like characteristics. These findings have major therapeutic implications. The selective targeting of specific types of pro-inflammatory macrophages and tailored immunomodulation of T-cell subsets may provide new strategies to stabilize plaques and other novel and targeted immunomodulatory approaches to prevent cardiovascular events. As single-cell and spatial technologies continue to evolve, they will further refine our ability to design precision immunotherapies for atherosclerosis by integrating classical inflammatory models with high-resolution molecular insights.

## Introduction

**Figure 1.**
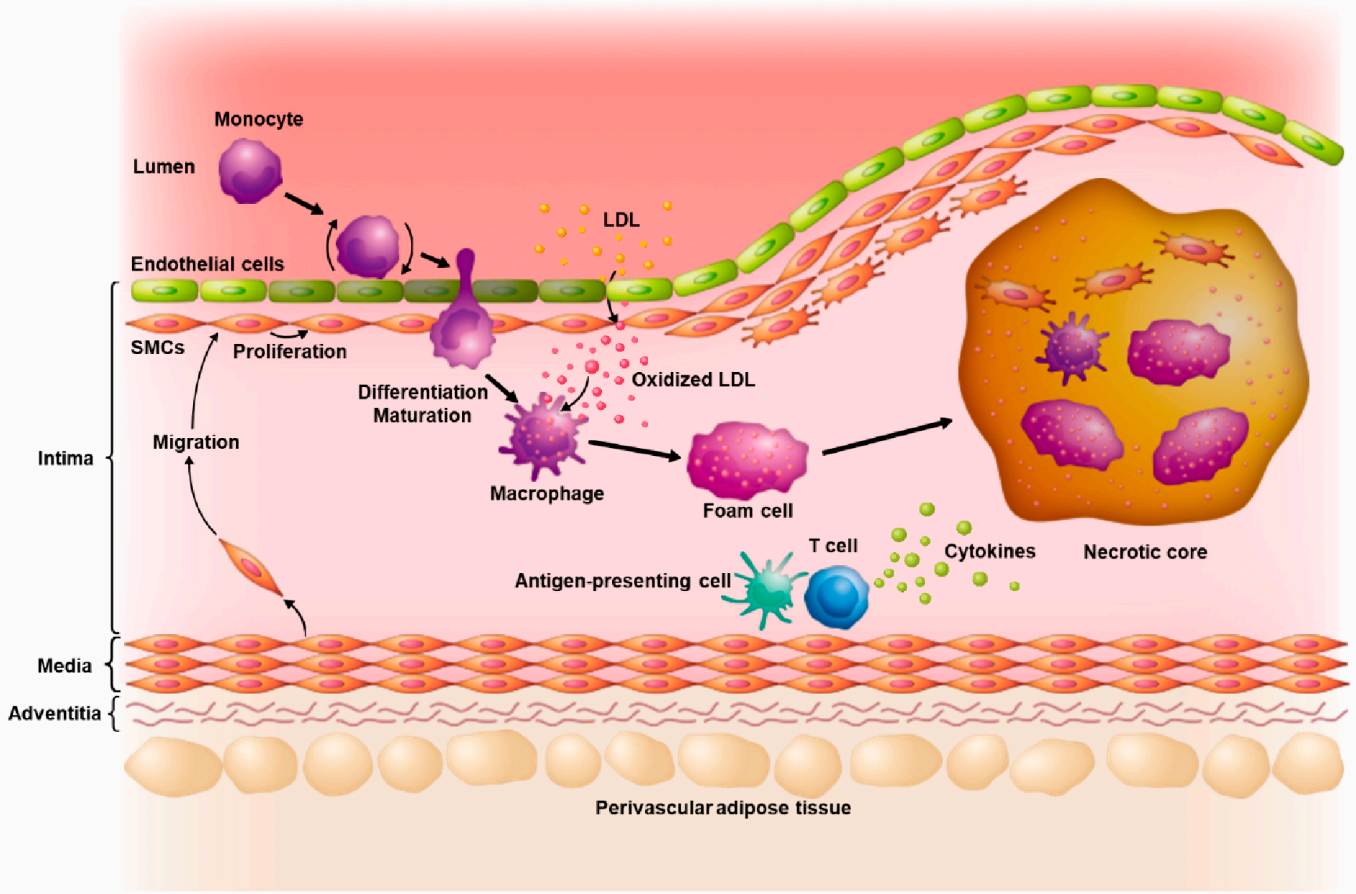
Classical model of atherosclerotic plaque development based on the response-to-injury hypothesis. Endothelial injury promotes monocyte recruitment and smooth muscle cell migration into the intima, where they proliferate. Monocytes differentiate into macrophages and foam cells on the uptake of oxidized LDL. T cells infiltrate the plaque and contribute to local inflammation by secreting cytokines and interacting with antigen-presenting cells. Perivascular adipose tissue surrounding the vessel may also influence plaque development through immune and paracrine signaling. LDL: low-density lipoprotein; SMC: smooth muscle cell.

**Figure 2.**
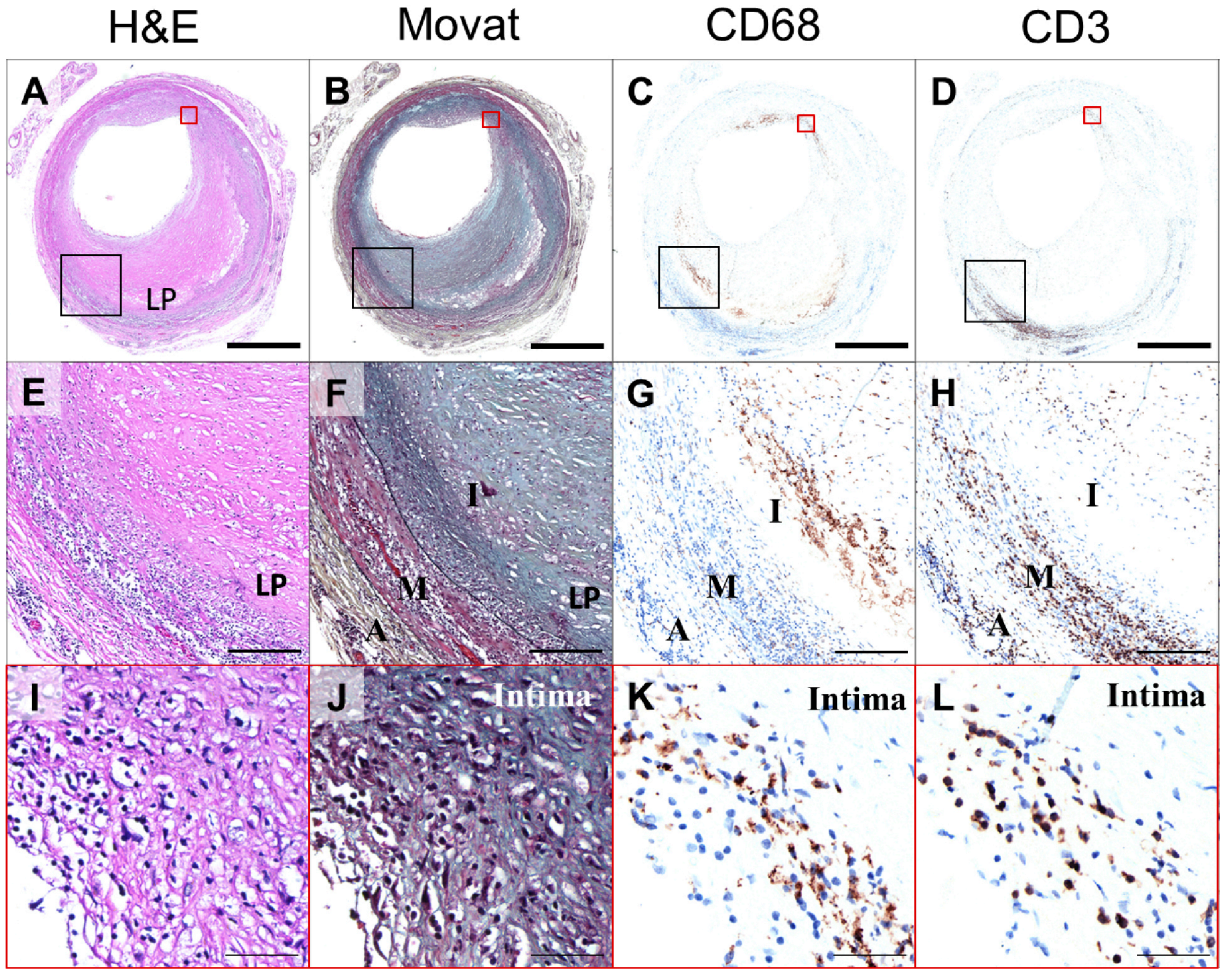
Macrophages and T cells are both abundant in human atherosclerotic plaque. (A) H&E section of an early fibroatheroma; the lipid-rich pool is labeled as LP within the intima. (B) Serial Movat Pentachrome section indicating the overall plaque architecture. (C) Immunohistochemistry against the pan-macrophage marker CD68 (brown). (D) Immunohistochemistry for the pan-T cell marker CD3 (brown). (E-H) Higher-magnification views of the larger black boxed areas in panels A-D, respectively, with labeled features indicated. (I-L) Higher-magnification views of the smaller red boxed areas in panels A-D, respectively (all located in the intima). (G, K) CD68 staining confirms that macrophages are distributed throughout the lesion but are most concentrated along the lipid-rich pool and the luminal side of the intima. (H, L) CD3 staining shows T cells dispersed throughout the plaque, with a predilection for the lipid-rich pool as well as the media and the adventitia. Scale bars: top row = 1 mm; middle row = 200 μm; bottom row = 50 μm. A: adventitia; H&E: hematoxylin and eosin; I: intima; LP: lipid-rich pool; M: media.

Atherosclerosis―the underlying cause of coronary artery disease, stroke, and peripheral arterial disease―has long been recognized as more than a simple lipid storage disorder ^(1)^. According to the response-to-injury hypothesis proposed by Ross et al. ^(2)^, endothelial injury, followed by lipid and inflammatory cell insudation, promotes smooth muscle cells to migrate from the media into the intima, where they proliferate and contribute to plaque formation ^(2)^. This concept, later refined by Ross ^(3)^ in 1999, emphasized the central role of inflammation in atherogenesis. Subsequent research confirmed that the arterial wall mounts an immune response to various risk factors, such as dyslipidemia and hypertension, causing the recruitment of monocytes, T cells, and other leukocytes that drive chronic inflammation in plaques ^(4)^. Recent studies have recognized perivascular tissues as important components of the vascular structure that contribute to the regulation of vascular inflammation ^(5)^. This historical perspective helped establish a paradigm in which atherosclerosis is driven by maladaptive immune responses to vascular injury and lipid deposition (Figure 1). These insights have prompted efforts to define the immune cell subsets and mediators involved in atherogenesis. Classical immunohistochemistry, flow cytometry, and bulk RNA sequencing studies have identified macrophages and T lymphocytes as the key components of atherosclerotic lesions (Figure 2), with macrophage-derived foam cells and T-cell-derived cytokines playing critical roles in plaque development ^(6), (7), (8)^. However, these traditional approaches, which rely on bulk tissue analysis or predefined cell subsets or targets, lack the resolution to capture the full spectrum of cell states or identify rare but functionally important populations. Advancements in single-cell analysis have revolutionized our understanding of atherosclerosis by providing a detailed cellular atlas of plaques, first in murine models and later in human samples (summarized in Table 1) ^(9), (10), (11), (12), (13), (14), (15), (16), (17), (18), (19), (20), (21), (22), (23), (24), (25), (26), (27), (28)^. By delineating the immune cell subsets and activation states within human atherosclerotic lesions, single-cell studies hold great promise for identifying precise therapeutic targets within the immune compartment of plaques.

**Table 1.** Summary of Single-Cell and Spatial Transcriptomic Studies on Atherosclerotic Plaques.

Authors (year)	Samples (species, tissue)	Method	Key findings
Winkels et al. (2018) ^(9)^	Mouse (Apoe^−/−^, Ldlr^−/−^ aortic plaques)	scRNA-seq	Established an immune cell atlas of atherosclerotic aortas, identifying 11 distinct leukocyte clusters in Apoe^−/−^ and Ldlr^−/−^ mice.
Cochain et al. (2018) ^(10)^	Mouse (Ldlr^-/-^ model: normal vs. high-fat diet aortic plaques)	scRNA-seq	Identified 13 immune cell clusters, including inflammatory macrophages (*Il1b*^high^) and *Trem2*^high^ lipid-associated macrophages.
Wirka et al. (2019) ^(11)^	Mouse (Apoe^-/-^ aortic plaques with SMC-*Tcf21* knockout); human coronary arteries from explanted transplant hearts	scRNA-seq + SMC lineage tracing	Showed that SMCs do not transdifferentiate into macrophages but instead transition into fibroblast-like “fibromyocytes”. Higher *TCF21* expression correlated with greater fibromyocyte presence and reduced CAD risk, confirming TCF21 promotes SMC phenotypic modulation in atheroprotection.
Fernandez et al. (2019) ^(12)^	Human carotid plaques (patients with symptoms vs. those without)	scRNA-seq + CITE-seq	Symptomatic plaques contained activated CD4^+^ T cell subsets (high PD-1 expression) and alternative macrophages linked to plaque vulnerability, whereas asymptomatic plaques showed IL-1β pathway immune activation.
Alencar et al. (2020) ^(13)^	Human carotid plaques; mouse (Apoe^-/-^ plaques with SMC/EC lineage tracing)	scRNA-seq + dual-lineage tracing	Identified *Lgals*3^+^ pioneer cells, which transition into osteogenic states in advanced plaques. Knockout of *Klf4* blocked this transition, indicating that it plays a key role in late-stage plaque pathogenesis.
Pan et al. (2020) ^(14)^	Mouse (Ldlr^-/-^ SMC lineage-traced aortic plaques) + human plaques	scRNA-seq + lineage tracing	Discovered stem-endothelial-monocyte-like multipotent cells in atherosclerotic plaques, capable of differentiating into macrophage-like or fibrochondrocyte-like cells. Identified retinoic acid signaling as a key regulator, which, when pharmacologically activated, reduced plaque burden and stabilized the fibrous cap.
Depuydt et al. (2020) ^(15)^	Human carotid endarterectomy plaques	scRNA-seq + scATAC-seq	Identified 14 distinct cell clusters, highlighting immune cell heterogeneity. Integrated chromatin accessibility data revealed transcription factor networks driving cell state transitions and spatial microanatomy within plaques.
Newman et al. (2021) ^(16)^	Mouse (Apoe^-/-^ brachiocephalic plaques, SMC-*Pdgfrb* knockout) and human plaques	scRNA-seq + lineage tracing	Found that fibrous cap myofibroblasts in plaques are primarily derived from smooth muscle cells, whereas a subset originates from endothelial or macrophage lineages through endothelial-to-mesenchymal transition or macrophage-to-mesenchymal transition.
Cheng et al. (2022) ^(17)^	Mouse (SMC-specific *Zeb2* perturbation model); human coronary SMCs	scRNA-seq + scATAC-seq	Identified *ZEB2* as the causal gene at a CAD risk locus. Integrated transcriptomic and chromatin accessibility data revealed that ZEB2 regulates SMC phenotypic plasticity through epigenetic remodeling, promoting pro-inflammatory and osteogenic transitions in plaques.
Emoto et al. (2022) ^(18)^	Human coronary culprit plaques (ACS vs CCS patients)	scRNA-seq	Identified distinct immune profiles in ACS plaques, including increased monocytes, mast cells, and inflammatory macrophages with high *CXCL3* and *IL1B* expression, compared with CCS plaques.
Chowdhury et al. (2022) ^(19)^	Human coronary plaques	scRNA-seq + scTCR-seq	Identified clonally expanded CD8^+^ T cells in plaques with TCRs reactive to viral peptides that mimic self-antigens. Revealed cross-reactivity between viral and arterial peptides, suggesting molecular mimicry as a mechanism of T cell-driven vascular inflammation.
Wang et al. (2023) ^(20)^	Mouse (Apoe^-^/^-^ aorta with HFD); human carotid plaques	scRNA-seq + scTCR-seq	Showed breakdown of T cell tolerance checkpoints in atherosclerosis. Clonally expanded T cells in plaques showed enrichment for *PDCD1*, *CTLA4*, and pro-inflammatory genes, suggesting sustained antigenic stimulation.
Depuydt et al. (2023) ^(21)^	Human carotid plaques and paired blood	scRNA-seq + scTCR-seq	Revealed clonally expanded CD4^+^ effector memory T cells enriched in plaques with autoimmune-like transcriptional signatures. Identified CD69^+^FOS^+^ T cells indicative of recent TCR stimulation and tissue residency.
Sun et al. (2023) ^(22)^	Human carotid plaques	Spatial transcriptomics	Identified distinct transcriptional signatures in plaque areas prone to rupture, providing spatial insights into plaque vulnerability.
Tan et al. (2023) ^(23)^	Human carotid plaques and paired blood samples	scRNA-seq	Constructed a single-cell atlas of carotid atherosclerosis, identifying immune and endothelial subsets associated with cerebrovascular events. Highlighted pro-inflammatory T cell subsets and monocyte/macrophage states enriched in symptomatic plaques, linking immune heterogeneity to plaque vulnerability.
Mori et al. (2024) ^(24)^	Human carotid plaques	scRNA-seq	CD163^+^ macrophages trigger NF-κB-dependent EndMT within the fibrous cap; *CD163* deletion attenuated EndMT and plaque growth, and an EndMT cluster enriched for apoptosis genes was identified by single-cell profiling.
Bashore et al. (2024) ^(25)^	Human carotid plaques	scRNA-seq + CITE-seq	Identified multiple cell populations and their subtypes in human plaques. Enhanced understanding of cell heterogeneity and interactions, informing mechanisms underlying plaque progression.
Gastanadui et al. (2024) ^(26)^	Human coronary plaques	Spatial transcriptomics	Revealed that inflammatory gene expression profiles differed between stable and unstable coronary plaques, highlighting spatially resolved mechanisms of plaque stability.
Bleckwehl et al. (2025) ^(27)^	Human coronary and carotid atherosclerotic plaques (various stages)	Spatial transcriptomics + scRNA-seq	Created a spatial transcriptomic atlas of atherosclerotic plaques, revealing that microvascular remodeling is linked to immune cell recruitment and plaque progression. Identified specific immune-microvascular niches as potential therapeutic targets for plaque stabilization.
Lai et al. (2025) ^(28)^	Human carotid endarterectomy plaques; paired perivascular adipose tissue and blood	Spatial transcriptomics + scRNA-seq	Identified tertiary lymphoid-organ-like niches marked by fibroblast-like SMCs expressing *CXCL12*, *CCL19*, and *VCAM1*; these niches recruit lymphocytes and are independently associated with symptomatic carotid disease.

ACS: acute coronary syndrome; *Apoe^-^/^-^*: apolipoprotein E knockout; CAD: coronary artery disease; CCS: chronic coronary syndrome; CITE-seq: cellular indexing of transcriptomes and epitopes by sequencing; EC: endothelial cell; EndMT: endothelial-to-mesenchymal transition; HFD: high-fat diet; *Ldlr^-^/^-^*: low-density lipoprotein receptor knockout; PD-1: programmed cell death protein 1; scATAC-seq: single-cell assay for transposase-accessible chromatin sequencing; scRNA-seq: single-cell RNA sequencing; scTCR-seq: single-cell T cell receptor sequencing; SMC: smooth muscle cell; TCR: T cell receptor.

This review provides a comprehensive overview of recent advances in single-cell analysis of atherosclerotic plaques. First, we summarize the state-of-the-art single-cell technologies in atherosclerosis research and their applications. We then discuss key biological insights, particularly the diversity of macrophage and T-cell populations revealed in plaques. Finally, we consider the therapeutic implications of these findings, exploring ways targeting specific immune cells or their interactions could improve clinical outcomes. Through this analysis, we illustrate ways single-cell technologies are transforming our understanding of atherosclerosis, linking classical concepts such as the response-to-injury hypothesis with cutting-edge plaque biology to advance cardiovascular therapy.

## Recent Advances in Single-Cell Technologies for Atherosclerosis

Single-cell and spatial omics technologies have enabled researchers to dissect the cellular composition of atherosclerotic lesions with unprecedented resolution. Later, we outline several key platforms―single-cell RNA sequencing (scRNA-seq), single-cell assay for transposase-accessible chromatin sequencing (scATAC-seq), cellular indexing of transcriptomes and epitopes by sequencing (CITE-seq), single-cell T-cell receptor (TCR) sequencing (scTCR-seq), and spatial transcriptomics―and highlight their applications in atherosclerosis research.

### scRNA-seq

**Figure 3.**
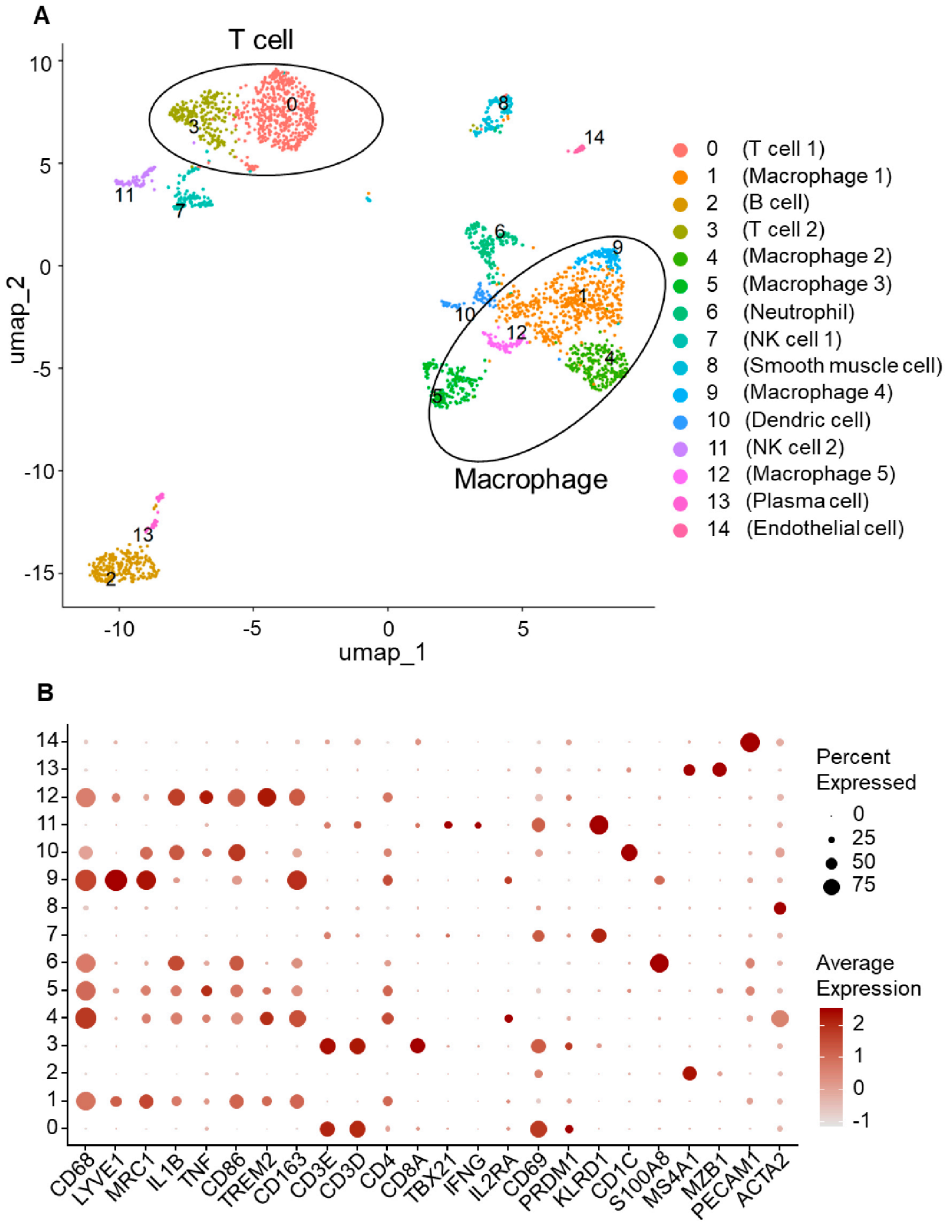
Single-cell RNA sequencing reveals diverse immune cell populations, including macrophages and T cells, in human atherosclerotic plaque. (A) UMAP projection generated from reanalysis of single-cell RNA-seq data from human coronary artery plaques (dataset GSE131778) ^(11)^. For this analysis, only *PTPRC* (CD45)-positive leukocytes were extracted and visualized, highlighting immune cell diversity within the lesion. Macrophage clusters are the most abundant, followed by T cell clusters. (B) Dot plot showing the expression of selected marker genes (x-axis) across individual clusters (y-axis) as defined in panel A. Dot size represents the percentage of cells expressing each gene in the cluster, and color intensity reflects average expression level.

This technique profiles gene expression in individual cells, allowing the unbiased identification of cell types and states based on transcriptomic signatures. Pioneering studies in mouse models first applied scRNA-seq to bulk-sorted aortic leukocytes from *Apoe*^-/-^ and *Ldlr*^-/-^ mice, revealing previously unrecognized subsets of macrophages, T cells, dendritic cells, and other immune populations ^(9), (10)^. The approach was soon extended to human specimens. Wirka et al. ^(11)^ conducted the first single-cell transcriptomic study of human coronary arteries, using explanted transplant hearts and focusing primarily on smooth muscle cells (SMCs), which they found to undergo phenotypic modulation into fibroblast-like “fibromyocytes”. In our reanalysis of this dataset, we specifically extracted *PTPRC* (CD45)-positive leukocytes to focus on immune cell diversity. Figure 3 illustrates the result of this reanalysis, showing that macrophages remain the most abundant immune cell cluster, followed by a substantial population of T cells. Subsequent scRNA-seq studies in human carotid endarterectomy and coronary atherectomy specimens have confirmed that many of the immune subsets originally identified in mouse lesions have clear analogues in human plaques ^(12), (13), (15), (18), (19), (21)^. Collectively, scRNA-seq has become a cornerstone technology for mapping the cellular landscape of atherosclerotic lesions across species in an unbiased and high-resolution manner. However, its reliance on tissue dissociation and limited capture efficiency for rare or fragile cells remain significant limitations.

### scATAC-seq

scATAC-seq profiles open chromatin regions at the single-cell level, enabling the identification of active regulatory elements such as promoters, enhancers, and transcription factor binding motifs. In atherosclerosis research, this technique has been instrumental in elucidating the epigenetic basis of immune cell function within plaques. For example, studies combining scATAC-seq with scRNA-seq in human carotid plaques revealed that macrophages and T cells exhibit distinct chromatin accessibility signatures that correlate with their transcriptional states and effector functions ^(15), (17)^, highlighting separate regulatory circuits for cytokine signaling and lipid uptake. Although each nucleus provides only a sparse snapshot of accessible sites, and the assay captures neither DNA methylation nor histone modifications, scATAC-seq, when combined with complementary methods, still offers unique insights into the transcriptional control mechanisms that drive atherosclerosis.

### CITE-seq

CITE-seq combines scRNA-seq with the simultaneous measurement of cell-surface proteins through DNA-barcoded antibodies. This technology is particularly useful in atherosclerosis studies for refining immune cell phenotyping, given many leukocyte subsets are defined by surface marker expression. In the context of plaques, CITE-seq has been applied to paired blood and plaque samples, linking mRNA-based clusters to well-known immune cell types through surface protein markers. Fernandez et al. ^(12)^ used CITE-seq in human carotid plaques, profiling up to 30 surface proteins along with transcriptomic data. This approach allowed a precise distinction between different T-cell subpopulations, such as CD4^+^ effector memory T cells vs. regulatory T cells (Tregs), based on *FOXP3* transcript levels and CD25 protein expression. Moreover, CITE-seq validated the presence of distinct macrophage subsets, differentiating CD206^high^ from CD206^low^ macrophages, and confirmed that CD8^+^ T cells and CD163^+^CD206^+^ “M2-like” macrophages are enriched in plaques relative to blood ^(12)^. By integrating gene expression with immunophenotyping, CITE-seq enhances our ability to define immune cell states within plaques with greater resolution than does transcriptomics alone.

### scTCR-seq

scTCR-seq identifies paired TCR α and β chains at the single-cell level. In atherosclerosis, it has revealed clonally expanded T cells within plaques and linked TCR clonotypes to gene expression profiles ^(20), (21)^. Expanded CD4^+^ and CD8^+^ clones often show activation or exhaustion markers such as proto-oncogene c-Fos (FOS) and programmed cell death protein 1 (PD-1), suggesting chronic antigen stimulation ^(20), (21)^. scTCR-seq has also provided evidence of molecular mimicry and antigen-driven responses, offering insights into adaptive immunity in vascular inflammation ^(19)^.

### Spatial transcriptomics

One limitation of dissociative single-cell methods is the loss of spatial context―i.e., the inability to determine the precise locations of cells within plaque architecture (e.g., fibrous cap, necrotic core, and shoulder regions). Spatial transcriptomics addresses this by profiling gene expression in intact tissue sections while preserving positional information. Although resolution is often limited to small cell clusters (rather than true single-cell resolution), spatial transcriptomic platforms can be paired with adjacent immunostained sections to assist in cell type annotation. Recent spatial transcriptomic studies of human atherosclerosis have provided insights into ways cell populations are organized within plaques and ways this organization differs between stable and unstable lesions. For example, Sun et al. ^(22)^ used spatial transcriptomics on human carotid plaques and identified region-specific gene expression patterns associated with plaque rupture versus stable areas. Rupture-prone regions showed enrichment of pathways related to matrix metalloproteinases and heightened inflammation, whereas more stable regions showed signatures of smooth muscle cell matrix deposition that contribute to fibrous-cap thickening and plaque stabilization ^(22)^. Gastanadui et al. ^(26)^ applied spatial profiling to coronary plaques from patients with acute coronary syndromes vs. stable angina. The authors found that unstable plaques showed significant upregulation of pathways related to interferon-γ (IFN-γ) and tumor necrosis factor-α (TNF-α), whereas stable plaques exhibited a transcriptional program more consistent with vascular wall homeostasis, findings likely to promote lesion stability ^(26)^. Bleckwehl et al. ^(27)^ recently generated a comprehensive spatial transcriptomic atlas of human coronary plaques, highlighting microvascular remodeling and immune cell recruitment patterns in different regions of the plaque. This study identified distinct immune-microvascular niches, defined as localized microdomains where microvessels and immune cells, such as macrophages and lymphocytes, are spatially co-localized and engage in cellular interactions that contribute to plaque progression. Their findings suggest that targeting these specific microvascular niches may offer therapeutic strategies for stabilizing high-risk plaques ^(27)^. Extending these findings, Lai et al. ^(28)^ applied spatial transcriptomics and scRNA-seq to human carotid endarterectomy specimens and uncovered tertiary lymphoid-organ-like immune niches marked by fibroblast-like SMCs expressing *CXCL12*, *CCL19*, and *VCAM1* that recruit lymphocytes and are associated with plaque instability. Although spatial transcriptomic data are highly dependent on tissue RNA quality and currently provide near, rather than true, single-cell resolution, they nonetheless complement dissociative single-cell sequencing approaches by enabling researchers to map the spatial localization of specific cell phenotypes within the complex plaque microenvironment.

In summary, the advent of single-cell technologies has provided powerful tools for cataloging the cellular constituents of atherosclerotic plaques and probing their molecular states. In the following sections, we discuss key biological insights gained from these approaches, with a focus on two major themes: macrophage diversity and T-cell heterogeneity in plaques, both of which have been central to recent discoveries in single-cell studies.

## Macrophage Diversity in Atherosclerotic Plaques

**Figure 4.**
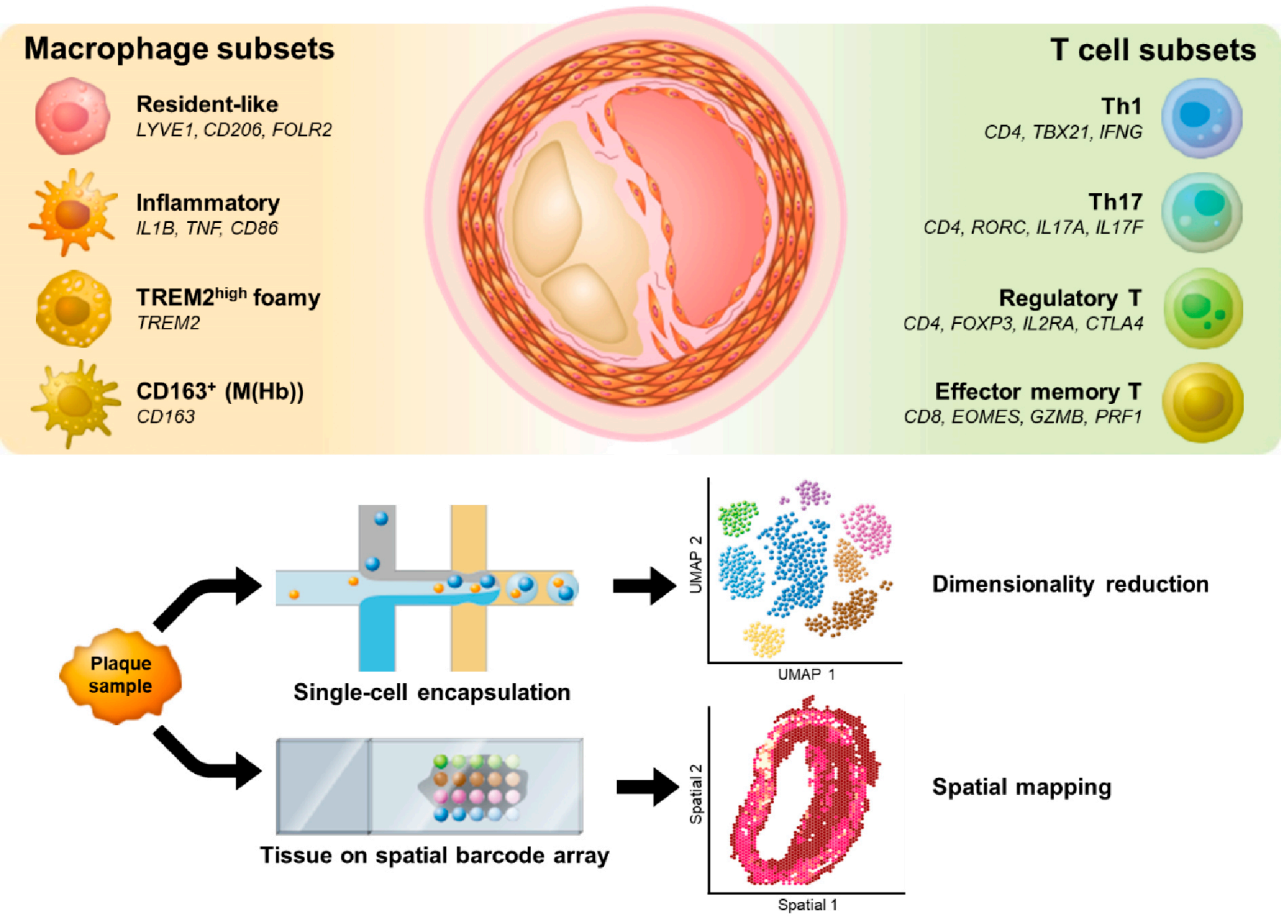
Single-cell and spatial transcriptomic profiling reveals immune cell heterogeneity in atherosclerotic plaques. Single-cell and spatial transcriptomic analyses revealed that macrophages and T cells are the predominant immune cell types within atherosclerotic plaques, each comprising multiple transcriptionally distinct subsets. Macrophages include resident-like populations (expressing representative markers such as *LYVE1, MRC1*, and* FOLR2*), inflammatory subsets (*IL1B*, *TNF*, *CD86*), *TREM2*^high^ foamy macrophages enriched for lipid-handling genes (*TREM2*), and CD163^+^ macrophages that adopt an M(Hb) phenotype after hemoglobin-haptoglobin exposure (*CD163*). CD4^+^ T cells are classified into Th1 (*TBX21*, *IFNG*), Th17 (*RORC*, *IL17A*, *IL17F*), and regulatory T cells (*FOXP3*, *IL2RA*, *CTLA4*), whereas CD8^+^ T cells include effector memory subsets (*EOMES*, *GZMB*, *PRF1*) and others. These immune populations exhibit distinct spatial distributions within the plaque, suggesting region-specific immune responses that may contribute to lesion progression or stability.

Macrophages are the predominant immune cells in atherosclerotic lesions (Figure 2 and 3), playing critical roles in lipid uptake, inflammation, plaque progression, and tissue remodeling. Single-cell analyses have identified a heterogeneous spectrum of macrophages, including resident-like, inflammatory, and triggering receptor expressed on myeloid cells 2 (TREM2)^high^ foamy subsets, in addition to a CD163^+^ subset that encompasses the hemoglobin-stimulated (M(Hb)) phenotype (Figure 4). This heterogeneity is not captured by the classical M1/M2 paradigm; instead, transcriptomic profiling now classifies plaque macrophages into these broad functional states.

### Resident-like macrophages

A subset of lesional macrophages exhibits a gene profile similar to that of resident tissue macrophages, such as those derived from embryonic progenitors or normally residing in the arterial intima. These cells typically express homeostatic genes and scavenger receptors involved in debris clearance. In murine aortas, Cochain et al. ^(10)^ identified an arterial resident-like macrophage cluster characterized by high expression of *Lyve1*, *Mrc1* (Cd206), *Folr2*, *Gas6*, and *F13a1* and by low levels of inflammatory cytokine transcripts. Fernandez et al. ^(12)^ identified a subset of CD206^high^ macrophages in human plaques, consistent with this resident/repair phenotype. Subsequent integrated single-cell analyses have confirmed a corresponding cluster of macrophages expressing *LYVE1* and *MRC1* in human plaques whose transcriptome overlaps with murine resident macrophages ^(29)^. In the arterial wall, these resident-like macrophages are found predominantly in the adventitia and to a lesser extent in the intima ^(30), (31), (32)^. They constitute the principal macrophage population in non-atherosclerotic arteries, originating largely from embryonic yolk-sac progenitors, and are maintained through local proliferation ^(30), (31), (32), (33)^. However, under the conditions of sustained lipid accumulation, even resident-like macrophages can become lipid-loaded, potentially altering their function and contributing to lesion progression ^(34)^.

### Inflammatory macrophages

A distinct subset of plaque macrophages is characterized by the high expression of pro-inflammatory genes, including *IL1B* and *TNF* (in humans), and various chemokines, in addition to inflammatory surface markers such as *Ly6c* (in mice). These cells resemble “classically activated” M1 macrophages and are often derived from newly recruited monocytes. In single-cell analyses, Cochain et al. ^(10)^ identified an *Il1b*^high^ inflammatory macrophage cluster present exclusively in murine atherosclerotic aortas. In human plaques, a corresponding “M1-like” inflammatory macrophage subset has been identified, distinguished by markers such as *IL1B* and *TNF* ^(12), (15)^. Such cells likely drive lesion progression by secreting classical pro-inflammatory cytokines, which amplify local inflammation and recruit additional leukocytes. Notably, inflammatory macrophages are implicated in plaque vulnerability because they produce proteases that degrade fibrous-cap collagen and tissue factors that promote thrombosis. Studies using single-cell and spatial transcriptomic approaches have shown that inflammatory macrophages are enriched in rupture-prone plaque regions. For instance, spatial transcriptomic analysis revealed that genes such as *TNF* and *MMP9* are upregulated in macrophage-dense regions of ruptured plaques, suggesting their involvement in extracellular matrix degradation and plaque destabilization ^(22), (26)^. Targeting pathways that are active in this subset (such as interleukin [IL]-1β signaling) is a promising therapeutic approach, as evidenced by the Canakinumab Anti-inflammatory Thrombosis Outcomes Study (CANTOS), in which IL-1β inhibition led to fewer cardiovascular events ^(35)^, presumably by attenuating IL-1β-mediated inflammation from plaque macrophages, among other anti-inflammatory mechanisms.

### TREM2^high^ foamy macrophages

Recent single-cell analyses of atherosclerotic lesions have identified a distinct subset of lipid-engorged, “foamy” macrophages marked by the high expression of *TREM2* in both mice and humans ^(10), (15)^. These cells exhibit a unique transcriptional profile characterized by genes involved in lipid uptake, cholesterol metabolism, and tissue repair, while showing a limited expression of classical inflammatory cytokines. This phenotype suggests that TREM2^high^ macrophages may buffer local inflammation and support tissue remodeling in advanced plaques. However, accumulating evidence suggests that the role of these cells is context-dependent and may differ between stages of atherogenesis. In early lesions, TREM2 signaling promotes the uptake of oxidized low-density lipoprotein (LDL) and supports macrophage survival and proliferation, thereby expanding the foam cell pool and accelerating plaque growth. In line with this, myeloid-specific TREM2 deletion reduces plaque size owing to impaired lipid accumulation and increased macrophage apoptosis ^(36)^. In contrast, in advanced plaques, TREM2^high^ macrophages exert protective effects. They facilitate efferocytosis and limit necrotic core formation, which is a key feature of unstable plaques. In this context, TREM2 deficiency causes impaired efferocytosis, increased necrosis, and plaque destabilization ^(37)^, whereas re-expression of TREM2 in myeloid cells restores macrophage function and promotes fibrous-cap stability in mouse models ^(38)^. Consistently, higher levels of soluble TREM2 in patients with advanced atherosclerosis reflect the accumulation or activation of TREM2-expressing macrophages in later-stage lesions ^(37)^. Overall, current evidence suggests that TREM2^high^ macrophages do not represent a single phenotype but rather exist along a continuum of lipid-laden states, highlighting TREM2 as an attractive yet complex therapeutic target.

### CD163^+^ macrophages and their M(Hb) subset

Originally considered atheroprotective, recent single-cell studies have redefined CD163^+^ macrophages―often termed M(Hb) macrophages when conditioned by hemoglobin-haptoglobin (HH) complexes―as key drivers of plaque progression after intraplaque hemorrhage (IPH). On stimulation by HH at IPH sites, these cells upregulate *VEGFA*, *HIF1A*, and matrix-remodeling genes, promote the formation of leaky neovessels, exacerbate endothelial activation and inflammatory cell recruitment, and―through crosstalk with vascular smooth muscle cells―limit calcification, thereby maintaining plaque vulnerability ^(39), (40)^. Mechanistic studies further indicate that M(Hb) macrophages secrete factors inducing nuclear factor-κB-dependent, pro-apoptotic endothelial-to-mesenchymal transition (EndMT) within the fibrous cap. Consistent with these findings, scRNA-seq of human carotid plaques has identified an EndMT cluster enriched for apoptosis-related genes. Moreover, deletion of *Cd163* in *Apoe*^-^/^-^ mice attenuated EndMT, preserved cap thickness, and slowed plaque progression ^(24)^. Thus, M(Hb) macrophages represent a distinct, HH-activated sub-lineage that links IPH to angiogenesis, EndMT, and fibrous-cap destabilization ^(41)^.

## T-Cell Heterogeneity and Function in Atherosclerosis

Recent single-cell studies indicate that although murine atherosclerotic lesions remain largely myeloid-dominant, human plaques harbor a comparatively larger T-cell compartment that can approach―or in some cohorts account for―up to half of all infiltrating leukocytes ^(11), (12)^ (Figure 3). T cells are distributed throughout plaques, including the lipid pool, media, and adventitia (Figure 2), where they interact with antigen-presenting cells ^(42)^. In this review, we focus on major T-cell clusters―including T helper (Th)1, Th17, Tregs, CD8^+^ T cells, and tissue-resident memory T cells (T_RM_)―which have been implicated in the pathogenesis of atherosclerosis through a combination of experimental studies and recent insights from single-cell analyses (Figure 4).

### Th1 cells

Th1 cells are the most abundant CD4^+^ T-cell subset in atherosclerotic plaques, and they play a central pro-atherogenic role ^(12)^. They are defined by the high expression of *TBX21* and *IFNG*, and produce IFN-γ. Th1 cells activate macrophages and endothelial cells, enhance inflammatory chemokine production, and promote plaque instability ^(43), (44)^. Single-cell studies confirm that many plaque-infiltrating CD4^+^ T cells exhibit a Th1 gene signature, reinforcing their contribution to atherogenesis ^(12)^.

### Th17 cells

Th17 cells are a less abundant subset of CD4^+^ T cells, defined by *RORC* expression and production of IL-17A/F ^(45)^. These cells recruit neutrophils and monocytes and amplify local inflammation. However, IL-17 may also contribute to fibrous-cap formation in some contexts, suggesting that Th17 cells exert context-dependent effects in atherosclerosis ^(46), (47)^. The precise role of Th17 cells in human plaques remains an area of active investigation.

### Tregs

Although Tregs constitute only a small fraction of CD4^+^ T cells within atherosclerotic plaques, they exert potent atheroprotective effects ^(23), (48)^. Transcriptionally defined by the high expression of *FOXP3*, *IL2RA* *(CD25)*, and *CTLA4*, Tregs suppress Th1 and Th17 responses and secrete anti-inflammatory cytokines such as IL-10 and TGF-β ^(49)^. Experimental studies have shown that Treg expansion enhances plaque stability, whereas Treg depletion accelerates disease progression ^(49), (50), (51)^. Moreover, single-cell analyses have revealed that symptomatic plaques exhibit a higher Th1/Treg ratio than do asymptomatic lesions, suggesting an imbalance between Th1 and Tregs may contribute to plaque vulnerability ^(23)^.

### CD8^+^ T cells

CD8^+^ T cells are abundant in advanced human atherosclerotic plaques ^(12)^. Traditionally, CD8^+^ T cells function as cytotoxic lymphocytes that target virus-infected or tumor cells; however, in atherosclerosis, they may also kill vascular cells, including endothelial cells, SMCs, and macrophages that present self-antigens. Single-cell analyses have identified two major CD8^+^ T-cell phenotypes in plaques: an effector memory-like population (*EOMES*^+^, *GZMB*^+^, *PRF1*^+^, low *CCR7*), which exhibits strong cytotoxic potential, and an exhausted population, characterized by the co-expression of inhibitory receptors (*PDCD1*, *CTLA4*, *HAVCR2*, *LAG3*), indicative of chronic antigen exposure and functional exhaustion ^(12)^. Functionally, activated CD8^+^ T cells in plaques secrete IFN-γ and TNF-α, amplifying local inflammation. Cytotoxic activity against SMCs may contribute to fibrous-cap weakening, increasing the risk of plaque rupture. Conversely, T-cell exhaustion, characterized by *PDCD1* (PD-1) expression, may suppress excessive inflammation. However, reversal of this exhausted state, for example, through PD-1 blockade in cancer immunotherapy, has been associated with accelerated atherosclerosis, suggesting that CD8^+^ T-cell activity must be tightly regulated ^(52), (53)^. Single-cell analyses further indicated that CD8^+^ T cells in plaques exist along a spectrum of activation and exhaustion, underscoring the complex role of adaptive immunity in lesion progression ^(12)^.

### T_RM_

T_RM_ cells are a fraction of plaque T cells that persist in non-lymphoid tissues, including atherosclerotic plaques, without recirculating through the bloodstream. They are characterized by the expressions of CD69 and CD49α, markers associated with their long-term retention in tissues. Recent studies have shown that T_RM_ cells contribute to atheroprotection by modulating the local immune environment, reducing intralesional macrophage content, and enhancing plaque stability ^(54)^. In murine models, the depletion of T_RM_ cells, achieved through the deletion of key transcription factors *Hobit* and *Blimp-1*, generated increased macrophage accumulation and decreased collagen deposition within plaques, leading to reduced lesion stability ^(54)^. This suggests that T_RM_ cells may exert protective effects by suppressing chronic inflammation and promoting a more stable plaque phenotype ^(54)^.

## Atherosclerosis as an Autoimmune-Like Disease: Rationale and Evidence

Although traditionally considered a lipid-driven disorder, increasing evidence supports the notion that atherosclerosis also exhibits autoimmune-like features. One of the key advances supporting this concept is the application of scTCR-seq to atherosclerotic lesions. This technology enables the identification of clonally expanded T-cell populations with defined transcriptomic profiles, a hallmark of antigen-driven immune responses. In both human and murine studies, scTCR-seq has revealed restricted TCR diversity and oligoclonal expansions within atherosclerotic plaques, indicating that local T cells are not bystanders but have been activated and expanded in response to specific antigens ^(19), (20), (21)^.

Among the best-characterized candidate antigens is apolipoprotein B (ApoB), the core protein component of LDL. ApoB accumulates in the arterial intima during early atherogenesis, when it may undergo oxidative modification and be presented by antigen-presenting cells. Several studies have identified ApoB-reactive CD4^+^ and CD8^+^ T cells in humans and mice, and many of these clones are clonally expanded within plaques ^(21), (55), (56)^. Functional assays, including major histocompatibility complex class II tetramer staining and T-cell proliferation assays, have revealed that these T cells recognize ApoB-derived peptides and acquire pro-inflammatory phenotypes, suggesting a breakdown of immune tolerance to this self-antigen ^(56), (57), (58), (59)^.

The presence of expanded, antigen-specific T cells that recognize a modified self-protein, such as ApoB, aligns with immunological features observed in classical autoimmune diseases ^(19), (21), (55), (56), (59)^. Furthermore, the similarity in T-cell clonal architecture in atherosclerosis and autoimmune conditions such as psoriatic arthritis provides additional evidence for a shared immunopathogenic mechanism ^(60), (61)^.

In addition to ApoB, recent studies suggest that prior exposure to viral antigens may also drive T-cell expansion through molecular mimicry ^(19)^. For instance, virus-specific CD8^+^ T cells have been shown to cross-react with structurally similar self-peptides expressed in the arterial wall, potentially contributing to chronic vascular inflammation ^(21)^. Taken together, these findings suggest that self-antigens and infection-related antigens may shape the T-cell repertoire in plaques.

The concept of atherosclerosis as an autoimmune-like disease helps explain the persistence of inflammation in plaques despite lipid-lowering therapy and provides a rationale for immunomodulatory approaches. Strategies aimed at restoring immune tolerance, such as ApoB-specific tolerizing vaccines or Treg-based therapies, are currently under investigation ^(62), (63)^. As our understanding of plaque-associated T-cell specificity deepens, antigen-targeted immunotherapies may represent a novel frontier in the treatment of atherosclerotic cardiovascular disease.

## Therapeutic Targeting of Immune Pathways

A major goal of mapping the immune landscape of atherosclerotic plaques is to identify therapeutic strategies that selectively modulate pathogenic immune components while preserving protective elements. The success of anti-cytokine therapies, such as IL-1β neutralization in the CANTOS trial ^(35)^ and IL-6 inhibition in subsequent clinical studies ^(64), (65), (66), (67)^, underscores the potential of targeted anti-inflammatory interventions in reducing cardiovascular events but also the risks of targeting major inflammatory pathways in a non-selective manner. Patients in the treatment had increased risks of fatal infections or sepsis compared with control. Low-dose colchicine, which inhibits activation of the NOD-, LRR-, and pyrin domain-containing protein 3 (NLRP3) inflammasome and reduces IL-1β, was approved by the Food and Drug Administration in 2023 for secondary prevention of coronary artery disease, and recent reviews and meta-analyses collectively suggest that it provides a modest but clinically meaningful reduction in recurrent cardiovascular events, despite some heterogeneity across studies ^(68), (69), (70), (71)^. Single-cell analyses provide valuable insights for immune-targeted therapies in atherosclerosis. For instance, the identification of *IL1B*^high^ macrophages within plaques suggests that selectively attenuating this inflammatory subset could suppress most disease-driving immune cells ^(10)^. Conversely, enhancing Treg functions through IL-2 therapy or immune checkpoint agonists could stabilize plaques by restraining excessive Th1 and Th17 cell activity ^(72)^. The single-cell discovery of *PDCD1*^high^ exhausted T cells in human plaques ^(12)^ presents challenges and opportunities. Although T-cell exhaustion naturally limits chronic inflammation, reversing this state through interventions such as PD-1 blockade in cancer immunotherapy could inadvertently exacerbate atherosclerosis ^(52), (73)^. This highlights the need for selective immune modulation, given broad immune activation may have unintended cardiovascular consequences. Taken together, this integrative framework is guiding the development of next-generation immunomodulators that aim to disarm pathogenic immune circuits in atherosclerosis while preserving, or even enhancing, protective elements. However, achieving precise and cell-specific targeting remains a critical challenge.

## Translational Perspectives from Single-Cell Plaque Biology

The application of single-cell analysis to atherosclerosis is transforming our conceptual understanding of the disease. Although earlier models depicted a relatively linear process of lipid deposition and macrophage foam cell formation, modern research reveals a more dynamic and complex interplay among various immune cell subsets within evolving plaque microenvironments. A key advancement in this field is the integration of single-cell discoveries with the foundational response-to-injury hypothesis. Originally proposed by Ross et al. ^(2)^, this hypothesis suggests that vascular injury triggers an immune-inflammatory cascade, ultimately leading to lesion formation ^(2), (3), (8)^. Single-cell data have refined this perspective by identifying specific immune cell subsets and elucidating their functional roles within this cascade. These findings affirm that innate and adaptive immunity act in concert, but they also reveal that the immune response in atherosclerosis is not a uniform inflammatory reaction but rather a network of multiple immune modules, some promoting disease whereas others counteract it. The inflammation-proliferation cycle proposed by Ross is supported by single-cell analyses, which show that macrophages proliferate within plaques in response to inflammatory cues ^(10), (15), (25)^. At the same time, these studies reveal homeostatic immune mechanisms, such as immune checkpoint expression and Treg-mediated suppression, that were not accounted for in the original hypothesis ^(9), (10), (12)^. As a result, the classical response-to-injury model has evolved into a more dynamic network model of atherogenesis, in which diverse immune cell subsets and molecular pathways participate in complex feedback and regulatory loops that shape disease progression ^(9), (10)^.

From a therapeutic perspective, perhaps the most significant implication of single-cell research is the validation of immune pathways as drug targets. The CANTOS trial, which successfully showed that IL-1β inhibition reduced cardiovascular events ^(35)^, supports the concept that targeting inflammatory pathways can provide clinical benefit but also significant risks. Single-cell studies explain this success, revealing that a significant fraction of plaque macrophages are *IL1B*^high^ and drive local inflammation ^(18)^. Recent single-cell findings suggest that immune-modulating therapies targeting key inflammatory pathways, such as IL-1 or IL-6, may hold promise in stabilizing atherosclerotic plaques ^(20), (74)^. Furthermore, antigen-specific T-cell responses, particularly those involving ApoB-reactive clones, could provide novel therapeutic targets. Strategies such as peptide-based tolerance therapy or immune checkpoint modulation are under investigation, with single-cell TCR profiling offering new insights into their potential impact on plaque-associated T cells ^(19), (20), (21)^. Together, these insights position single-cell plaque biology as a critical driver of future atherosclerosis research and lay the foundation for precision medicine, which remains experimental because no immune-targeted agent has yet been approved specifically for this disease.

### Conclusions

Recent advances in single-cell analysis have provided unprecedented resolution in mapping the immune landscape of atherosclerosis. These technologies have not only reinforced the central role of inflammation but have also uncovered new layers of immune regulation within plaques. By bridging classical theories, such as the response-to-injury hypothesis, with molecular-level discoveries, single-cell studies have reshaped our understanding of atherogenesis. Moreover, these insights are actively driving the development of next-generation therapies designed to modulate immune responses, stabilize plaques, and prevent cardiovascular events. As single-cell and spatial transcriptomic technologies continue to advance, they will enhance our understanding of ways immune cell subsets contribute to disease progression and resolution.

## Article Information

This article is based on the study, which received the Medical Research Encouragement Prize of The Japan Medical Association in 2024.

### Authors Contributions

Yusuke Adachi conducted the literature search and drafted the manuscript. Alyssa Grogan, Rika Kawakami, Tatsuya Shiraki, Teruo Sekimoto, Takamasa Tanaka, Kazuhiro Fujiyoshi, Takafumi Nakayama, Tomoyo Hamana, Desiree Williams, Keisha Medina Diaz, Renu Virmani, and Aloke V. Finn critically reviewed and revised the manuscript. All authors read and approved the final version of the manuscript.

### Conflicts of Interest

CVPath Institute has received institutional research support from Leducq Foundation, Abbott Vascular, Ablative Solutions, Absorption Systems, Advanced NanoTherapies, Aerwave Medical, Alivas, Amgen, Asahi Medical, Aurios Medical, Avantec Vascular, BD, Biosensors, Biotronik, Bolt Medical, Boston Scientific, EndoVascular, Chansu Vascular Technologies, Children’s National, Concept Medical, Cook Medical, Cooper Health, Cormaze, CRL, Croivalve, CSI, Dexcom, Edwards Lifesciences, Elucid Bioimaging, eLum Technologies, Emboline, Endotronix, Envision, Filterlex, Innovalve, Innovative Cardiovascular Solutions, Intact Vascular, Interface Biologics, Intershunt Technologies, Invatin, Lahav, MedAlliance, Medanex, Medtronic, Mercator, Microvention, Neovasc, OrbusNeich, Pi-Cardia, Polares Medical, Polyvascular, Profusa, Protembis, Pulse Biosciences, Recor Medical, Shockwave, SMT, SoundPipe, Spectrawave, Surmodics, Terumo Corporation, The Jacobs Institute, UCSF, UPMC, Vascudyne, and Xeltis outside the submitted work. Dr. Finn has received honoraria from Abbott Vascular; Boston Scientific; Cook Medical; and is a consultant to Abbott Vascular; Medtronic, Cordis, and Boston Scientific; and Cook Medical outside the submitted work. Dr. Virmani is a consultant/scientific advisory board member of Abbott Vascular; Boston Scientific; CeloNova; Cook Medical; CSI; Edwards Lifesciences; Bard BD; Medtronic; OrbusNeich Medical; ReCor Medical; SinoMedical Sciences Technology; Surmodics; Terumo Corporation; W. L. Gore; and Xeltis. The other authors declare no competing interests.

### Ethical Approval

Human atherosclerotic specimens illustrated in this review were obtained from the CVPath Institute Registry under protocols approved by the institutional review board of CVPath Institute. All tissues were de-identified before analysis; therefore, the board granted a waiver of informed consent. The use of autopsy material was classified as non-human-subjects research and complied with the Declaration of Helsinki and applicable U.S. regulations.
